# VOC Emission Screening of Consumer Products in Microchambers: Comparison of Online PTR-MS and Offline TD–GC–MS Analysis

**DOI:** 10.1021/acs.analchem.5c04892

**Published:** 2026-02-20

**Authors:** Luise Klein, Helen Haug, Andreas Stenzel, Jonathan Beauchamp, Alexander Roloff

**Affiliations:** † Department of Chemical and Product Safety, 27652 German Federal Institute for Risk Assessment (BfR), Berlin 10589, Germany; ‡ Institute of Chemistry, Technical University Berlin, Berlin 10623, Germany; § Department of Sensory Analytics and Technologies, 28469 Fraunhofer Institute for Process Engineering and Packaging IVV, Freising 85354, Germany; ∥ Department of Chemistry and Pharmacy, Chair of Aroma and Smell Research, Friedrich-Alexander-Universität Erlangen-Nürnberg, Erlangen 91054, Germany

## Abstract

Emissions of volatile organic compounds (VOCs) from consumer products into the indoor air can lead to the inhalation exposure of consumers to potentially hazardous chemicals. This investigation compared two analytical approaches to quantitatively screen VOC emissions from consumer products. A microchamber was used to examine the emissions of two selected products—a rain poncho and a skipping rope—under controlled conditions (temperature, air change rate), with a focus on the initial short-term emissions. Volatiles were sampled from the chamber either on sorbent tubes, with subsequent offline analysis by thermal desorption–gas chromatography–mass spectrometry (TD-GC-MS), or with online analysis by proton transfer reaction–mass spectrometry (PTR-MS). The products emitted several compounds of toxicological relevance, including cyclohexanone, xylene, phenol, isophorone, and naphthalene. Data from the two approaches yielded similar emission curves for most compounds, with maximum concentrations observed within 30 min. Isophorone exhibited the highest peaks, with concentrations of ∼1.2 mg m^–3^ and ∼1.6 mg m^–3^ from TD-GC-MS and PTR-MS analysis, respectively. Discrepancies in the maximum concentrations between the methods ranged between 25% and 50% for most of the investigated compounds. Total amounts of emitted compounds were calculated as an alternative metric to single concentration values, which allow the assessment of inhalation exposure. This study demonstrates the utility of the two approaches in characterizing short-term VOC emissions from consumer products. PTR-MS analysis offers rapid quantitation of VOCs but has limitations in identifying individual compounds, highlighting the need for complementary analyses by TD-GC-MS. Despite inherent offline sampling, TD-GC-MS was shown to be well-suited to monitoring short-term emissions.

## Graphical abstract

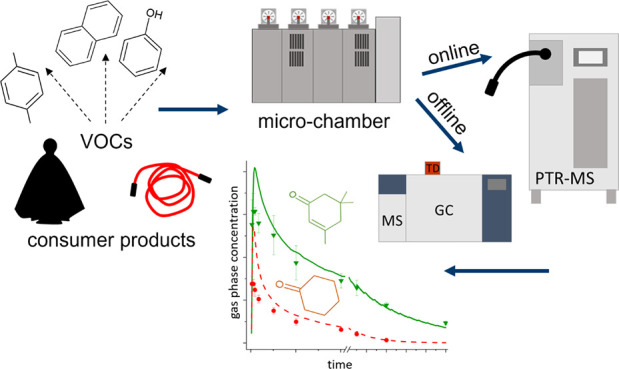

## Introduction

Consumer products, including toys, decorative articles, or textiles, are abundant in most households. These products can emit a broad variety of volatile organic compounds (VOCs) into the indoor environment at varying concentrations and consequently contribute to the exposure of consumers to potentially hazardous constituents.^1–3^ Inhalation of certain VOCs may elicit adverse effects, such as headaches or irritations of the eyes and nose, or induce or exacerbate respiratory conditions.^4–8^ Furthermore, some consumer products emit unpleasant smells, which not only present a nuisance but may be indicative of poor product quality in general, or specifically of the presence of potentially harmful volatiles.^9–12^ The European Union has established regulations to protect humans from exposure to VOCs, including specifying limits for the lowest concentration of interest (LCI), e.g., in the building sector for long-term emissions from construction products. These compound-specific guideline values have been derived based on the assumption of long-term exposure to steady gas-phase emissions of chemicals released into the ambient air from relevant materials.^13,14^ European and international standards, such as EN 16516 or ISO 16000-9, describe methods to screen for and quantify VOC emissions from construction products and furniture, allowing for a comparison of measured VOC emissions with established reference values.^15,16^

Volatile emissions from consumer products typically decline with age of the product as they gradually deplete from the source materials after their production. Unlike construction materials, which are installed in homes for long-term use, the exposure toward volatile emissions from consumer products may be particularly acute when the product is new, i.e., within the first hours or days after purchase and unpackaging. This is especially relevant in view of contemporary consumer habits of impulse purchasing and the associated high turnover of such products, leading to ever-changing emission sources of VOCs in the indoor environment. Accordingly, determining the initial, short-term peak emissions under realistic conditions of handling and use is necessary to evaluate acute inhalation exposure and estimate associated risks. This requires suitable analytical methods.^17^

Existing methods to determine long-term VOC emissions from materials using large-scale emission test chambers are generally not suitable for routine testing of small products, as has been recently reviewed.^17^ Large-scale emission test chambers have a sizable laboratory footprint, require high investment capital and are costly to run. Furthermore, single-sample test capabilities add to the limitations of these testing devices.^17^ An alternative to large-scale chambers are small-scale test chambers, commonly referred to as microchambers, that comprise vessels with small volumes (tens of milliliters) that allow for sampling smaller products or material pieces under controlled conditions.^9,18–20^

Previous studies have assessed the applicability of microchambers for characterizing VOC emissions from different consumer products.^19–22^ A targeted study on the emissions of 14 VOCs spiked into a polymer reference material investigated the comparability of their emissions in test chambers of different volume (44 mL, 24 L, and 203 L), whereby VOCs were intermittently sampled on Tenax TA-loaded sorbent tubes and subsequently analyzed by thermal desorption-gas chromatography–mass spectrometry (TD-GC-MS) over a 28-day period. The study found that the area-specific emission rates derived from the different test chambers were comparable after a few hours, irrespective of the chamber size. Since the study focused on overall compatibility between the chambers, however, with the VOC emission rates based on sampling periods of 30 min, the reported data do not provide insights into the concentration changes occurring within the first hour of emissions from new products.^20^

Characterizing the emission kinetics of VOCs from newly manufactured products requires alternative measurement strategies that allow for more frequent or continuous sampling. One suitable technology to achieve this is proton transfer reaction-mass spectrometry (PTR-MS), which is a direct injection technique that facilitates quantitation of gas-phase VOCs in real time.^23,24^ The technique is particularly suited to targeted analysis, when the compounds under consideration are known *a priori*, e.g., through complementary analysis by GC-MS to provide supporting information on compound identities.^25–27^

The suitability of PTR-MS to characterize emissions has been demonstrated in several test chamber studies. In one investigation, PTR-MS was used to monitor the drying process of water-based paints within the first hours after application, whereby the concentration of the compound trimethylamine released into the chamber air was quantified in parallel to TD-GC-MS analysis, with good alignment observed between the two approaches.^28^ In another experiment, the peak concentration of toluene emissions from a reference material in a 0.25 m^3^ emission test chamber was determined using PTR-MS and TD-GC-MS, with concentrations obtained by the two analytical techniques showing high deviations at the initial peak but a closer alignment with increasing sampling time. Furthermore, PTR-MS was shown to be better suited to characterize the peak concentrations in the initial phase of emissions compared to cumulative sampling on sorbent tubes.^28^

A recent study proposed coupling a microchamber to PTR-MS to characterize the emissions kinetics of VOCs from small objects.^29^ This approach allowed for a rapid and targeted analysis of emissions, especially for highly volatile compounds, as demonstrated by investigating VOCs released from 3D-printed cubes. While comprehensive TD-GC×GC-time-of-flight (TOF)-MS was employed to support the identification of compounds detected by PTR-MS, neither quantitation via TD-GC-MS nor a comparison of the emission kinetics derived from the two methods was undertaken.^29^ In a companion study, emission rates from 3D-printed cubes determined using the microchamber-PTR-MS setup were compared to inhalation exposure limits,^30^ demonstrating the potential of this approach for risk assessment.

Direct comparisons of analytical techniques for quantifying VOC emissions from different consumer products are required to assess their suitability in producing reliable data relevant to realistic exposure scenarios. This paper reports on the analysis of the early phase of VOC emissions from representative consumer products by TD-GC-MS and PTR-MS coupled to a microchamber. VOC emissions for TD-GC-MS analysis were sampled by collection on Tenax TA at frequent intervals whereas PTR-MS analysis proceeded continuously through direct sampling from the chamber. A quantitative comparison of these complementary approaches for analyzing emissions from consumer products is presented here for the first time. Both methods were appraised for their suitability to screen potentially harmful VOC emissions from consumer products, with a focus on short-term, initial peak emissions.

## Experimental Section

### Samples

Two consumer products – a rain poncho and a children’s plastic skipping rope – were selected for the analysis of their VOC emissions. The samples represent commercial products that were drawn from the German market by a State Office for further analysis on account of their strong and unpleasant odors, which may correlate with poor product quality and increased emissions of problematic VOCs.^9–12^ The poncho was cut into 1 cm × 1 cm square pieces, which were placed into the microchamber with the water-repellent outer surface facing upward (emitting surface area: 1 cm^2^). The skipping rope, with a diameter of 5 mm, was cut into 2 cm long cylindrical pieces (emitting surface area: 3.14 cm^2^), with the cut edges covered with aluminum tape to suppress the emissions from these freshly exposed surfaces. Pretests on the aluminum tape did not detect emissions of compounds relevant to the product. All samples were stored at room temperature in sealed, airtight aluminum bags until immediately before analysis.

### Microchamber Experiments

Two commercial small-scale emission test chamber units, referred to as microchambers (Micro-Chamber/Thermal Extractor – μ-CTE 250, Markes International Ltd., Bridgend, UK) consisting of four inert chambers each with 114 mL volume were used to characterize the VOC emission kinetics from the individual samples. The μ-CTEs were preconditioned at 150 °C for 30 min prior to the experiments. A flow of nitrogen gas at a rate of 100 mL min^–1^ (gas change rate: 52.6 h^–1^), which was the minimum flow required by the PTR-MS instrument, was applied to all chambers. Notably, this does not resemble realistic area-specific airflow rates, *q*, for such products but facilitated the comparability of data acquired from the two different analytical approaches. Three replicate samples of each product were placed individually into three separate chambers at a temperature of 23 °C (representing ambient temperature), resulting in loading factors, *L*, of 0.9 and 2.8 m^2^ m^–3^ for the poncho and skipping rope samples, respectively. After inserting the sample, the chambers remained closed throughout the experiments. The VOCs emitted from the products into the chamber were sampled directly at the respective chamber outlets during the first 12 h after loading, either via periodic sampling on Tenax TA sorbent tubes and subsequent TD-GC-MS analysis, or continuously by online PTR-MS analysis. These analyses were performed independently at different locations, with both samples first analyzed by PTR-MS and later (∼5 – 12 months interval) via TD-GC-MS.

### TD-GC-MS Analysis

The microchamber gas phase was sampled from the three loaded chambers in parallel by collection onto Tenax TA sorbent tubes (Gerstel GmbH & Co. KG, Mülheim an der Ruhr, Germany) periodically at various times. Sampling durations were 1–5 min, resulting in accumulated sampling volumes of 100–500 mL. Blanks were drawn from the chamber air prior to sample loading to ensure that background signals were below 5% of the analyte signals. Tubes for sampling and calibration were preconditioned at 300 °C for 3 h in a tube conditioner (TC 2, Gerstel GmbH & Co. KG) and subsequently spiked with 10 ng *p*-xylene-d_10_ as internal standard using a tube spiking system (TSS, Gerstel GmbH & Co. KG). The Tenax TA sorbent tubes were subsequently placed in a thermal desorption unit (TDU 2, Gerstel GmbH & Co. KG) coupled to a GC system (7890A, Agilent Technologies, Inc., Santa Clara, CA, USA). Volatile compounds were desorbed from the tubes and trapped in a cold injection system (CIS, Gerstel GmbH & Co. KG) prior to their focused injection in splitless mode into the GC. Analytes were separated on a DB-5MS column. VOCs were detected via mass spectrometry (5975C MSD, Agilent Technologies, Inc.) in combined scan/selected ion monitoring (SIM) mode over a range of *m*/*z* 29–450 using analyte-specific *m*/*z*-values of quantifier and qualifier ions (see Tables S1 and S2 in the Supporting Information, SI, for analytical details of the TD-GC-MS method). Compounds were identified by comparison of measured full-scan mass spectra to library reference spectra (NIST 20.L Mass Spectral Library, National Institute of Standards and Technology, U.S. Department of Commerce, Gaithersburg, MD, USA). Compound identities were confirmed by comparing retention times and mass spectra with those of measured reference standards. The detected SIM signals were used for quantitation of *o-/m-/p-*xylene (sum of isomers for comparability with PTR-MS data), cyclohexanone, 2-ethylhexanol, phenol, isophorone, and naphthalene via calibration (in relation to the *p*-xylene-d_10_ internal standard) with spiked standards in ethyl acetate within a working range of 5–160 ng. All standards were analytical grade, purchased from Sigma-Aldrich Corporation (St. Louis, MO, USA).

Data processing was performed with the MassHunter software (MassHunter Workstation, Quantitative Analysis, Version 10.2, Agilent Technologies, Inc.). Emission rates of individual volatiles were calculated by multiplying their concentrations (μg m^–3^) with the sampling flow rate (0.006 m^3^ h^–1^). The areas under the emission rate curves (*AUC*_*ER*_) were calculated using Origin (Version 2023, OriginLab Corporation, Northampton, MA, USA) to determine the total quantity of a compound being emitted over 12 h.

### PTR-MS Analysis

Online analysis by PTR-MS used a PTR-TOF 8000 instrument (IONICON Analytik GmbH, Innsbruck, Austria) that was directly coupled to the outlet of the loaded microchamber via a bespoke interface, as previously described.^29^ This allowed the product emissions in the chamber to be analyzed directly and continuously. The instrument was operated at a reduced electric field (*E*/*N*) of 138 Td (see Table S3 for further parameters); these conditions were chosen to provide good ionization efficiencies for a broad range of VOCs, as is a common approach in nontargeted analysis by PTR-MS. One replicate was analyzed continuously over 12 h, which indicated that the emission peak declined significantly within the first 3 h; consequently, the remaining two replicates were analyzed only for 3 h each. Blanks were measured for 10 min before each experiment to obtain background profiles of the empty chambers and ensure low contamination. Samples were then placed into the chambers (chamber-lids briefly opened, but remained closed thereafter) without interrupting the measurement.

Signal intensities of individual VOCs were recorded and averaged periodically over the 1 min data acquisition periods. Data were processed using the PTR-MS Viewer software (v3.4, IONICON Analytik GmbH). Raw signal intensities (counts per seconds, cps) were corrected for transmission. VOC concentrations (μg kg^–1^) were calculated by the software based on the H_3_O^+^ primary ion signal (isotopolog at *m*/*z* 21.022) and reaction coefficients, *k,* reported in the literature,^31^ or a standard coefficient^23,28^ if not available (Table S4). To correctly quantify each analyte, signals were corrected by their compound-specific branching ratios (Table S4), determined by analyzing reference standards, to account for fragmentation-induced signal losses. Phenol, cyclohexanone, the sum of xylene isomers (not differentiable by PTR-TOF-MS), naphthalene, and isophorone were quantified with their respective molecular ions. Analysis of 2-ethylhexanol showed a strong fragmentation into unspecific ions (mainly *m*/*z* 39.023), thus the dehydration product (*m*/*z* 113.132) was used for quantitation to reduce the likelihood of interferences, despite its lower abundance. After exporting the VOC concentration data (μg kg^–1^), mean blank values of the first 10 min of the empty chambers (below 5%) were subtracted from the concentration data. VOC gas-phase concentrations in the chamber air in μg m^–3^ were calculated by multiplying the concentrations in μg kg^–1^ with the density of nitrogen (1.251 kg m^–3^) as the carrier gas. The emission rates and total amounts of substances emitted (*AUC*_*ER*_) were determined using Origin 2023 (OriginLab Corporation), as described for the TD-GC-MS analysis.

## Results and Discussion

TD-GC-MS analysis of the poncho revealed several VOCs with high peak intensities (Figure S1). The highest signals were attributed to isophorone, cyclohexanone, and naphthalene, with lower concentrations observed for *o*-, *m*- and *p*-xylene (xylenes), phenol, and 2-ethylhexanol.

The emission profiles for these compounds (except 2-ethylhexanol) are presented in Figure 1. The emissions of all compounds show similar trends, with an increase to maximum concentrations, *c*_*max*_, within the first minutes, followed by a strong decrease over the next 2 h and a subsequent steady decrease until the end of analysis at 12 h. The profiles observed in the PTR-MS data were similar (Figure 1). Concentrations and standard deviations (SDs) of triplicate measurements of both methods at the sorbent tube sampling intervals are presented in Table S5. It should be noted that triplicate measurements by PTR-MS were limited to the first 3 h; the data from 3 to 12 h are derived from a single analysis.

**Figure 1:**
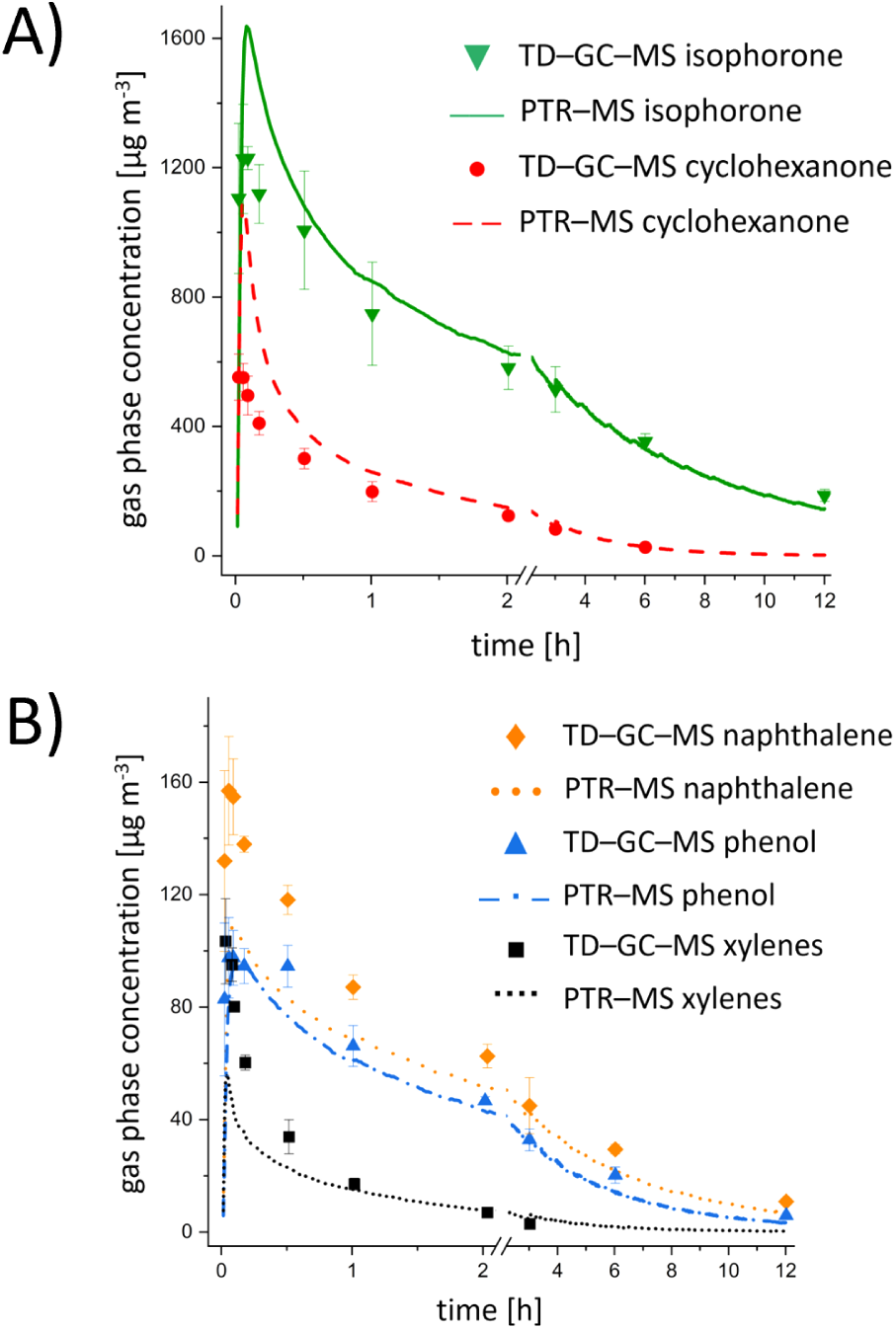
Emission profiles of the poncho sample over 12 h for (A) isophorone and cyclohexanone and (B) *o*-, *p*-, *m*-xylene (xylenes), phenol, and naphthalene as measured in a microchamber and detected via TD-GC-MS and PTR-MS. Xylenes and cyclohexanone were not quantifiable by TD-GC-MS at sampling time points beyond 3 h and 6 h, respectively.

Relative SDs of the replicates within the first minutes exhibited a maximum of 32.8% for phenol in TD-GC-MS and 27.4% for isophorone in PTR-MS. For TD-GC-MS analysis, the SDs decreased to 1.4–18.6% after 6–12 h (see Table S5 and Figure S2A). The lower SDs in the TD-GC-MS data at later sampling points could be attributed to the longer sampling durations and the associated reduced variation in sampling volume, which was necessary to achieve increased sensitivity. For PTR-MS, relative SDs were between 3.6% and 26.8% after 3 min and remained relatively consistent over the 3 h measurement period (see Table S5 and Figure S2B).

The main deviations between the data derived from the two methods were observed for *c*_*max*_ measured within the first hour (Figure S3). The value of *c*_*max*_ for cyclohexanone, for example, differed by a factor of approximately two between PTR-MS and TD-GC-MS analysis (1082.4 μg m^–3^ vs 552.6 μg m^–3^), respectively (Figure 2 and Table S6). The opposite discrepancy was observed for the sum of xylenes, whereby TD-GC-MS returned a higher concentration of 103.4 μg m^–3^ compared to 54.5 μg m^–3^ by PTR-MS (47.3% lower). The best alignment of peak concentrations was observed for phenol with 94.5 μg m^–3^ for PTR-MS and 97.5 μg m^–3^ for TD-GC-MS (3.1% lower via PTR-MS). The *c*_*max*_ values for isophorone (1637.3 μg m^–3^ and 1229.5 μg m^–3^) and naphthalene (108.4 μg m^–3^ and 156.9 μg m^–3^) derived from PTR-MS compared to TD-GC-MS analysis differed by +33.1% and −30.9%, respectively (see Figure 2).

**Figure 2:**
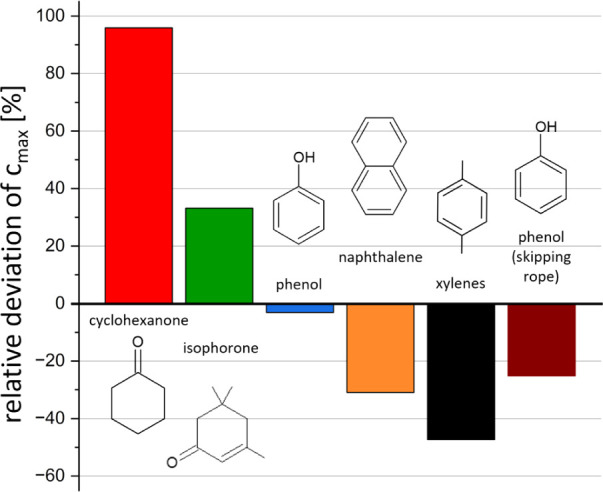
Relative deviations in the maximum compound concentrations (*c_max_*) derived from PTR-MS vs TD-GC-MS analysis. Deviations are calculated from the mean *c_max_* emitted from the poncho and skipping rope (see Table S6). Positive and negative values represent higher and lower estimations of *c_max_* by PTR-MS compared to TD-GC-MS.

The skipping rope emitted phenol and 2-ethylhexanol at notable concentrations, the latter albeit observed only in the TD-GC-MS data sets (Figure S4). The emission profiles of phenol from the skipping rope (Figure S5A) were similar to those of the poncho, with *c*_*max*_ of 56.4 μg m^–3^ (±24.4%) and 42.2 μg m^–3^ (±31.8%) within the first minutes, as determined by TD-GC-MS and PTR-MS, respectively (25.1% lower with PTR-MS), followed by a rapid decrease.

The emission curves of 2-ethylhexanol from both samples were different, with PTR-MS yielding significantly lower concentrations than TD-GC-MS (see Figures S5B and S6). The likely underestimation by PTR-MS could be attributable to the fragmentation of this compound upon ionization into less specific fragments under the applied operating conditions and an associated uncertainty in the branching ratio for the chosen *m*/*z*. Although lower *E/N* conditions in PTR-MS analysis have been demonstrated to be more suitable for detecting alcohols,^32^ the nontargeted approach of the present study dictated the use of a fixed *E/N* setting to facilitate an efficient detection of a broad range of compounds. Furthermore, the compounds of interest were selected in posthoc analysis of the data, thus the contribution and relevance of 2-ethylhexanol was not known *a priori* and consequently, its detection was not prioritized.

In general, the emission profiles obtained via the two methods aligned well for both samples and for five of the six compounds (except 2-ethylhexanol), although higher deviations ranging between 25% and 47% were observed for *c*_*max*_ for some compounds (up to ∼100% for cyclohexanone). Similar observations have been made in previous studies, where the alignment between PTR-MS and TD-GC-MS analysis of toluene emissions from a reference film improved with time (no explicit values were reported for comparison).^28^

In the present study, the observed differences in concentrations might be explained by several factors. To start with, different quantitation approaches were applied for each method. Whereas TD-GC-MS data were quantified via calibration using analytical standards, PTR-MS data utilized calculations based on kinetic theory that relied on theoretical reaction coefficient values whose accuracy can be up to ∼50%;^33^ nevertheless, the coefficients used in the present calculations have been shown to closely match experimentally determined values.^31^ Furthermore, the presence of signal interferences can affect VOC quantitation by PTR-MS. Although PTR-TOF-MS allows for the differentiation of nominally isobaric compounds, isomeric molecular species or fragments cannot be discriminated, which potentially leads to an overestimation of compound concentrations. Additionally, although Tenax TA is a good all-round sorbent, varying ad- and desorption efficiencies between compounds may contribute to deviations in concentrations in TD-GC-MS analysis, yet the use of calibration with reference standards in the present study should minimize this effect.

Another factor of consideration is that the chamber concentrations, especially within the first minutes of experiments, could be affected by sample loading. In the PTR-MS approach, the analyses were continuous, with detection of compound emissions immediately after placing the sample in the chamber and closing the lid, whereas the first sampling point for TD-GC-MS was after 1 min. Additionally, minor deviations during manual sampling in the initial phase of emissions, characterized by rapid concentration changes, could have an impact on the results, including reproducibility. Moreover, TD-GC-MS and PTR-MS experiments were performed across a larger timespan, i.e., with PTR-MS analyses performed first, followed by TD-GC-MS analyses approximately 5 to 12 months later, during which period material- and compound-related changes might have occurred, leading to different emissions characteristics (despite the samples being stored in tightly sealed pouches). This might be further influenced by an inhomogeneous distribution of compounds in the sample materials, as is a realistic scenario for consumer products.

Despite some discrepancies, the concentrations determined for most compounds with both methods in two different laboratories agree within 30% for sampling time points 5 min after chamber loading and later. For comparison, round robin tests for emissions analyses employing reference materials report typical relative SDs of between 28% and 50%.^34,35^ The deviations in this study, in which experiments were performed at different locations using different analytical techniques up to 12 months apart, can therefore be considered comparable and within the ranges observed in other studies.

In addition to the kinetic profiles of VOC emissions, the total amount of a compound released over a defined period is an important factor in the context of risk assessment of consumer products, e.g., to calculate daily exposure or intake.^2,30,36^ This is particularly relevant for the initial phase of compound emissions from new products, when the concentrations typically change rapidly. Although relatively high gas change rates in the microchamber were employed to accommodate sampling requirements of the PTR-MS instrument and thus deviated from realistic area specific airflow rates, the total amounts of compounds being emitted over time were calculated by integrating emission rates (area under the curve of the emission rate −*AUC*_*ER*_) for all quantified compounds to demonstrate the applicability of the two approaches (Table S7). As an example, the emission rate and *AUC*_*ER*_ of phenol from the skipping rope are shown in Figure 3A (*AUC*_*ER*_ values of all quantified compounds emitted from the poncho are presented in Figure S7). As the emission rates decrease over time, *AUC*_*ER*_ increases more slowly after 3 h to 6 h for most compounds. Both methods yield similar *AUC*_*ER*_ profiles, and the observed deviations (Figure 3B) are comparable to those observed for the concentrations (cf. Figure S3). Determining total amounts of emitted compounds as a function of time facilitates exposure assessment for varying exposure scenarios.

**Figure 3:**
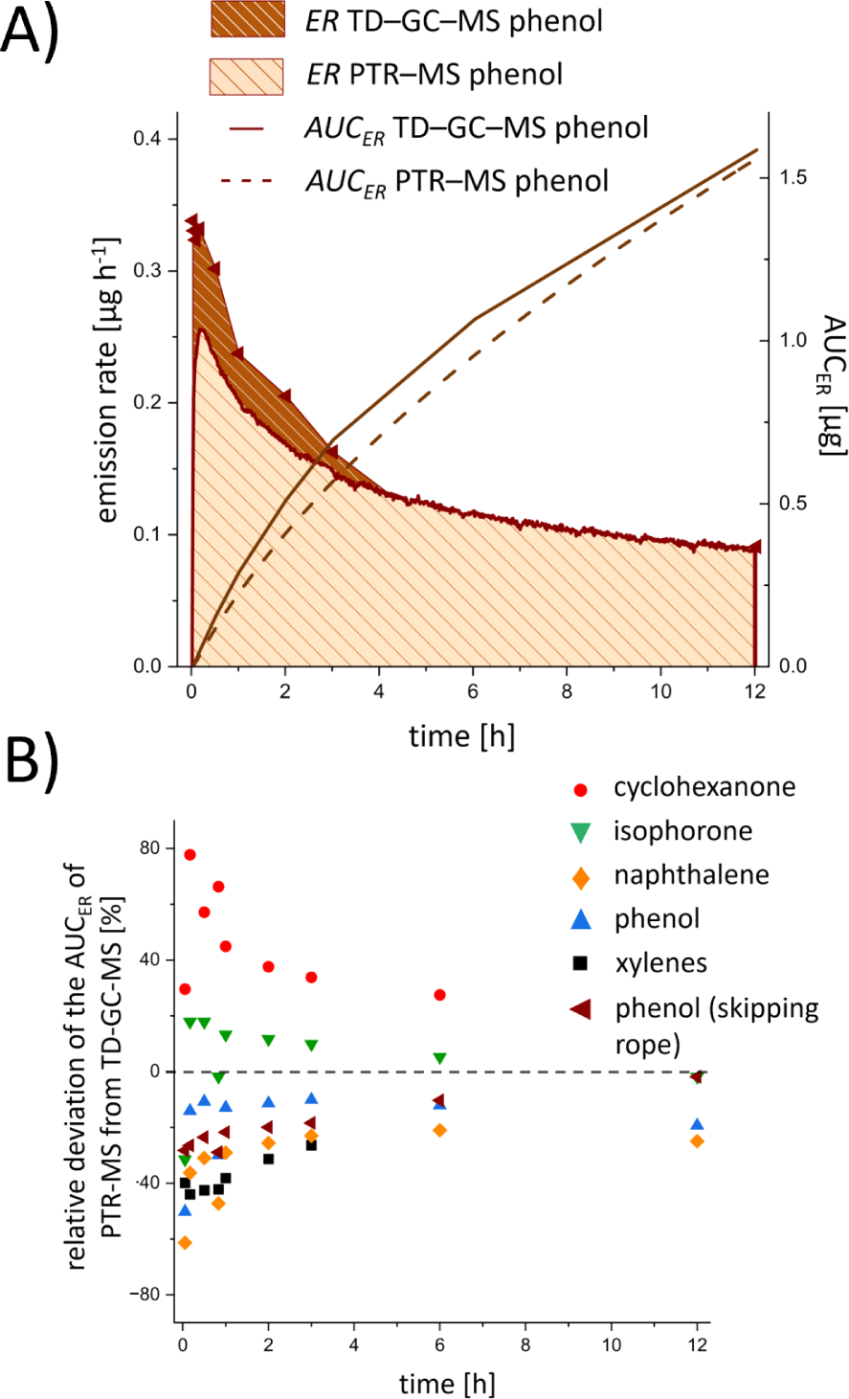
(A) Emission rates *ER* [μg h^–1^] of phenol from the skipping rope sample in a microchamber over 12 h (left *y*-axis), whereby integration of the area under the emission rate curve (*AUC_ER_*) provides the total amount of phenol emitted from the skipping rope (right *y*-axis). (B) Relative deviation of *AUC_ER_* values of emitted compounds from the poncho and skipping rope over 12 h determined via PTR-MS and TD-GC-MS (also see Table S7). Positive and negative values respectively represent higher and lower values determined by PTR-MS compared to TD-GC-MS.

Both analytical approaches were successfully applied to monitor the short-term, initial release of VOCs from consumer products through the use of microchambers. The major benefit of PTR-MS analysis is its capability for real-time measurements, which allows concentration changes in the gas phase to be detected with high temporal resolution, thereby making it especially suited to determining maximum concentrations during the initial emission stages. Additionally, the online sampling procedure requires minimal preparation, making microchamber-PTR-MS an easy-to-use method for emissions screening. The use of high-resolution mass spectrometers in combination with the soft ionization provided by PTR-TOF-MS can facilitate the identification of compounds due to accurate knowledge of element composition from the *m*/*z* signals. Nevertheless, when performing nontargeted or semitargeted screenings, identification and quantitation can be challenging (as presently observed for 2-ethylhexanol), requiring complementary GC-MS analysis to verify compound identities.

In comparison, TD-GC-MS offers accurate identification of unknown substances in untargeted screenings of VOC emissions and allows for subsequent quantitation via calibration. Covering maximum and rapid changes in concentrations can be challenging, however, and is only achievable by sampling frequently and rigorously during the initial phase of emissions, requiring additional effort in preparation and manual sampling. The sensitivity of this offline method depends on the sampling volume and, thus, sampling time. Extended sampling, however, reduces the sampling frequency and leads to averaging over longer periods during the initial emission phase, where an adequately high sampling frequency is crucial to allow for the assessment of short-term exposure peaks.

## Conclusions

There is a need to establish simple and quick screening methods to assess the emissions of volatiles from indoor sources for estimating inhalation exposure to harmful substances. This study demonstrates the use of microchambers combined with offline sorbent tube sampling and GC-MS analysis as well as with direct online PTR-MS analysis for first screenings of VOC emissions from consumer products, enabling conclusions to be drawn for further investigations and short-term exposures. Both approaches yielded similar VOC emission profiles for most quantified compounds. Automation and the ability to detect rapid concentration changes of VOC emissions with high temporal resolution suggest that online PTR-MS analysis may be the better suited technique to determine initial peak concentrations. Although tube sampling and TD-GC-MS analysis are commonly used for determining long-term emissions at quasi-equilibrium conditions, the present study highlights that this procedure is also suitable to reliably quantify VOC emissions during the early emission stage, when the concentrations of emitted compounds are typically still high. Additionally, determining emission rates and total amounts of emitted compounds as a function of time can be useful for exposure assessment, complementing the comparison of concentration values at single time points to reference values. Verification of observations for a broader range of consumer goods is necessary to validate the present findings for wider applications or prospective use in routine product screenings.

## Supplementary Material



## Notes

Tables summarizing additional analytical parameters, retention times and *m*/*z* values of compounds, gas-phase concentrations and *AUC*_*ER*_ values with standard deviations, and deviations in results obtained by both methods, as well as figures depicting chromatograms, emission profiles of phenol and 2-ethylhexanol, relative standard deviations of concentrations and *AUC*_*ER*_ over time (PDF)

## #

Bundesanstalt für Materialforschung und -prüfung, Berlin, Germany
