# Supplementary material for: VOC Emission Screening of Consumer Products in Microchambers: Comparison of Online PTR-MS and Offline TD–GC–MS Analysis
Source: Anal Chem. 2026 Feb 20;98(9):6468–74. doi: 10.1021/acs.analchem.5c04892 (PMC12980488; doi:10.1021/acs.analchem.5c04892)
Supplement: Supplementary file 1 [file ac5c04892_si_001.pdf]

Supporting Information

# VOC Emission Screening of Consumer Products in Micro-Chambers: Comparison of Online PTR-MS and Offline TD-GC-MS Analysis

*Luise Klein[a,b] †, Helen Haug[c,d], Andreas Stenzel[c,d], Jonathan Beauchamp[c,]*, Alexander Roloff[a,]**

[a] Department of Chemical and Product Safety, German Federal Institute for Risk Assessment (BfR), 10589 Berlin, Germany.

[b] Institute of Chemistry, Technical University Berlin, 10623 Berlin, Germany.

[c] Department of Sensory Analytics and Technologies, Fraunhofer Institute for Process Engineering and Packaging IVV, 85354 Freising, Germany.

[d] Department of Chemistry and Pharmacy, Chair of Aroma and Smell Research, Friedrich-Alexander-Universität Erlangen-Nürnberg, 91054 Erlangen, Germany.

## Abbreviations

$AUC_{ER}$ – area under the curve of the emission rate

CIS – cold injection system

$c_{max}$ – maximum concentration

PTR-MS – proton transfer reaction–mass spectrometry

PTR-TOF-MS – proton transfer reaction–time of flight–mass spectrometry

SD – standard deviation

SIM – selected ion monitoring

TD-GC-MS – thermal desorption–gas chromatography–mass spectrometry

TDU – thermal desorption unit

TIC – total ion chromatogram

$t_R$ – retention time

**Table of Contents**


**Table S1.** Retention times $t_R$ [min], mass-to-charge ratios (*m/z*) of the quantifier and qualifier ions and their relative responses of the analytes in the TD-GC-MS measurements. S-5

**Table S2.** Analytical parameters of the thermal desorption–gas chromatography–mass spectrometry (TD-GC-MS) method. S-6

**Table S3**. Analytical parameters of the proton transfer reaction–time of flight–mass spectrometry (PTR-TOF-MS) method. S-7

**Table S4**. Mass-to-charge ratios (*m/z*) of ions and associated multipliers used for the quantitation of the compounds in PTR-MS measurements. S-7

**Table S5**. Gas-phase concentrations and standard deviations (SDs) of the TD-GC-MS and PTR-MS measurements and deviation of PTR-MS values from TD-GC-MS values. S-9 – S-10

**Table S6**. Comparison of the maximum concentrations, $c_{max}$ [µg m$^{-3}$], and their relative deviations between the PTR-MS and the TD-GC-MS analyses. S-12

**Table S7**. Total quantities of emitted compounds (area under the emission rate curve – $AUC_{ER}$) [µg] and deviations between concentrations from PTR-MS and TD-GC-MS analysis. S-15 – S-16

**Figure S1.** TD-GC-MS chromatograms in selected ion monitoring (SIM) – mode (including qualifier and quantifier ions of all depicted analytes in the total ion chromatogram (TIC)) of the micro-chamber blank with the internal standard standard *p*-xylene-$d_{10}$ at $t_R$ = 10.3 min (top) and the poncho sample after 30 min in the micro-chamber (bottom). S-8

**Figure S2**. Relative SDs of compound concentrations from the poncho and skipping rope at different times as measured by A) TD-GCMS and B) PTR-MS. S-11

**Figure S3**. Relative deviations between the PTR-MS and TD-GC-MS analyses of the gas-phase concentrations of compounds emitted from the poncho and skipping rope over time. S-12

**Figure S4**. TD-GC-MS chromatograms in selected ion monitoring (SIM) – mode (including qualifier and quantifier ions of all depicted analytes in the total ion chromatogram (TIC)) of the micro-chamber blank with the internal standard standard *p*-xylene-$d_{10}$ at $t_R$ = 10.3 min (top) and the skipping rope sample after 30 min in the micro-chamber (bottom). S-13

**Figure S5**. Emission profiles of A) phenol and B) 2-ethylhexanol from the skipping rope sample in a micro-chamber over 12 h, as determined by TD-GC-MS and PTR-MS analysis. S-13

**Figure S6**. Emission profiles of 2-ethylhexanol from the poncho sample in a micro-chamber over 12 h, as determined by TD-GC-MS and PTR-MS analysis. S-14

**Figure S7**. Total quantity of emitted compounds (area under the emission rate curve – $AUC_{ER}$) [µg] from the poncho in a micro-chamber over 12 h, as determined by PTR-MS and TD-GC-MS.



## Supplemental Tables and Figures

**Table S1.** Retention times $t_R$ [min], mass-to-charge ratios (*m/z*) of the quantifier and qualifier ions and their relative responses of the analytes in the TD-GC-MS measurements.

| **compounds** | **retention time $t_R$ [min]** | ***m/z* quantifier ion** | ***m/z* qualifier ion 1** | **relative response [%]** | ***m/z* qualifier ion 2** | **relative response [%]** |
|---|---|---|---|---|---|---|
| *p*-xylene-$d_{10}$ | 10.34 | 98 | 114 | 48.4 | 116 | |
| *m*-,*p*-xylene | 10.41 | 91 | 105 | 24 | 106 | 51 |
| *o*-xylene | 10.82 | 91 | 105 | 20 | 106 | 48 |
| cyclohexanone | 10.88 | 55 | 42 | 65 | 69 | 32 |
| phenol | 11.82 | 94 | 66 | 31 | 65 | 23 |
| 2-ethylhexanol | 12.63 | 57 | 83 | 22 | 112 | 3 |
| isophorone | 14.39 | 82 | 54 | 12 | 138 | 20 |
| naphthalene | 15.75 | 128 | 102 | 9 | 129 | 12 |

**Table S2.** Analytical parameters of the thermal desorption–gas chromatography–mass spectrometry (TD-GC-MS) method.

| | |
|---|---|
| **TDU** | |
| Temperature program | |
| Initial *T* | 25 °C |
| Rate | 700 °C $min^{-1}$ |
| End | 280 °C for 2 min |
| Purge flow | 280 mL $min^{-1}$ |
| **CIS** | |
| Splitless | |
| Temperature program | |
| Initial *T* | -150 °C |
| Rate | 12 °C $s^{-1}$ |
| End | 285 °C for 15 min |
| **GC** | |
| Column | DB-5MS (60 m x 320 µm, 1 µm) |
| He-flow | 1.4 mL $min^{-1}$ |
| Oven temperature program | |
| Initial | 45 °C for 0.5 min |
| Rate 1 | 12 °C $min^{-1}$ |
| Hold | 200 °C for 5 min |
| Rate 2 | 20 °C $min^{-1}$ |
| End | 280 °C for 10 min |
| **MS** | |
| Solvent delay | 6.5 min |
| Temperatures | |
| Transfer line | 295 °C |
| Quadrupole | 150 °C |
| Ion source | 230 °C |
| Scan rate | 3.5 scan $s^{-1}$ |
| Scan range | *m/z* 15 – 450 |
| SIM | respective *m/z*-ions in Table S2 |

**Table S3.** Analytical parameters of the proton transfer reaction–time of flight–mass spectrometry (PTR-TOF-MS) method.

| **Inlet and drift tube** | |
|---|---|
| Inlet temperature, $T_{inlet}$ | 100 °C |
| Drift tube temperature, $T_{drift}$ | 80 °C |
| Drift tube pressure, $p_{drift}$ | 2.20 mbar |
| Drift tube voltage, $U_{drift}$ | 550 V |
| Extraction voltage, $U_{dx}$ | 34 V |
| Reduced electric field, *E/N* | 138 Td (1 Td = $10^{-21}$ V $m^{-2}$) |

**Table S4.** Mass-to-charge ratios (*m/z*) of ions and associated multipliers used for the quantitation of the compounds in PTR-MS measurements.

| **compound** | ***m/z* of the molecular ion** | ***m/z* of the fragment ion** | **branching ratio correction factor** | **reaction coefficient (*k*) [×$10^{-9}$ $cm^3$ $s^{-1}$]** |
|---|---|---|---|---|
| sum of xylenes | 107.086 | | 1.0 | 2.27 [1] |
| cyclohexanone | 99.080 | | 1.3 | 2.00 [2,3] |
| phenol | 95.048 | | 1.0 | 2.13 [1] |
| 2-ethylhexanol | | 113.132 | 14.1 | 2.00 [2,3] |
| isophorone | 139.112 | | 1.0 | 2.00 [2,3] |
| naphthalene | 129.070 | | 1.0 | 2.45 [1] |

[1] Cappellin, L.; Karl, T.; Probst, M.; Ismailova, O.; Winkler, P. M.; Soukoulis, C.; Aprea, E.; Märk, T. D.; Gasperi, F.; Biasioli, F. On Quantitative Determination of Volatile Organic Compound Concentrations Using Proton Transfer Reaction Time-of-Flight Mass Spectrometry. Environmental Science & Technology 2012, Vol. 46, Issue 4, 2283–2290, DOI: 10.1021/es203985t.
[2] Hansel, A.; Jordan, A.; Holzinger, R.; Prazeller, P.; Vogel, W.; Lindinger, W. Proton transfer reaction mass spectrometry: on-line trace gas analysis at the ppb level. Int. J. Mass Spectrom. Ion Processes 1995, 149–150, 609-619, DOI: 10.1016/0168-1176(95)04294-U.
[3] Schripp T.; Etienne S.; Fauck C.; Fuhrmann F.; Mark L.; Salthammer T. Application of proton-transfer-reaction-mass-spectrometry for Indoor Air Quality research. Indoor Air 2014, 24 (2), 178-189. DOI: 10.1111/ina.12061

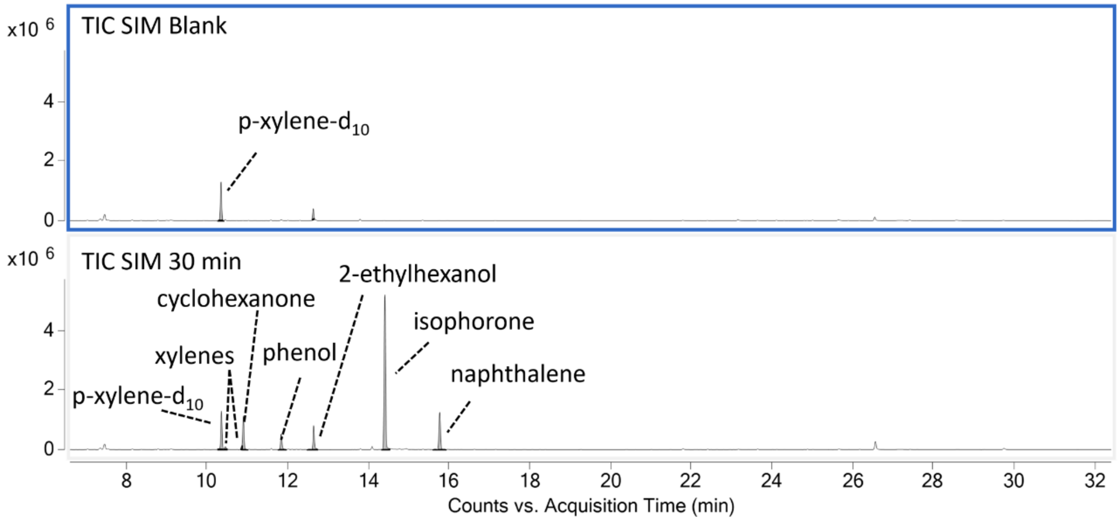


**Figure S1.** TD-GC-MS chromatograms in selected ion monitoring (SIM) mode (including qualifier and quantifier ions of all depicted analytes in the total ion chromatogram (TIC)) of the micro-chamber blank with the internal standard standard *p*-xylene-$d_{10}$ at $t_R$ = 10.3 min (top) and the poncho sample after 30 min in the micro-chamber (bottom).

**Table S5.** Gas-phase concentrations and standard deviations (SDs) of the TD-GC-MS and PTR-MS measurements and deviation of PTR-MS values from TD-GC-MS values.

| Sampling time | TD-GC-MS analysis | | | PTR-MS analysis | | | deviation of PTR-MS from TD-GC-MS | relative deviation of PTR-MS from TD-GC-MS |
|---|---|---|---|---|---|---|---|---|
| | concentration | SD | relative SD | concentration | SD | relative SD | | |
| | [µg m$^{-3}$] | [µg m$^{-3}$] | [%] | [µg m$^{-3}$] | [µg m$^{-3}$] | [%] | [µg m$^{-3}$] | [%] |
| **Poncho** | | | | | | | | |
| **compound** | **xylenes** | | | | | | | |
| 1 min | 103.41 | 15.10 | 14.61 | 42.04 | 11.00 | 26.16 | -61.37 | -59.35 |
| 3 min | 95.07 | 5.89 | 6.20 | 56.33 | 15.46 | 27.44 | -38.74 | -40.75 |
| 5 min | 80.18 | 0.41 | 0.51 | 50.05 | 13.43 | 26.83 | -30.13 | -37.58 |
| 10 min | 60.29 | 2.62 | 4.35 | 37.54 | 9.30 | 24.76 | -22.75 | -37.73 |
| 30 min | 33.90 | 6.03 | 17.78 | 23.65 | 6.11 | 25.86 | -10.25 | -30.24 |
| 1 h | 17.11 | 2.10 | 12.26 | 15.62 | 3.42 | 21.89 | -1.49 | -8.71 |
| 2 h | 6.93 | 0.14 | 2.05 | 8.03 | 2.07 | 25.75 | 1.10 | 15.87 |
| 3 h | 2.94 | 0.15 | 5.04 | 4.86 | 1.30 | 26.74 | 1.92 | 65.32 |
| 6 h | | | | 1.94 | | | 1.94 | |
| 12 h | | | | 0.34 | | | 0.34 | |
| **compound** | **cyclohexanone** | | | | | | | |
| 1 min | 552.55 | 71.45 | 12.93 | 822.86 | 124.03 | 15.07 | 270.31 | 48.92 |
| 3 min | 551.11 | 43.18 | 7.83 | 1092.49 | 191.70 | 17.55 | 541.38 | 98.23 |
| 5 min | 495.40 | 59.85 | 12.08 | 946.77 | 163.82 | 17.30 | 451.36 | 91.11 |
| 10 min | 409.73 | 36.25 | 8.85 | 682.49 | 106.01 | 15.53 | 272.76 | 66.57 |
| 30 min | 300.72 | 30.94 | 10.29 | 395.52 | 61.84 | 15.64 | 94.81 | 31.53 |
| 1 h | 198.30 | 30.60 | 15.43 | 259.29 | 32.05 | 12.36 | 60.99 | 30.76 |
| 2 h | 123.95 | 11.84 | 9.55 | 149.95 | 18.72 | 12.48 | 26.00 | 20.98 |
| 3 h | 82.39 | 6.16 | 7.47 | 93.78 | 12.53 | 13.36 | 11.39 | 13.83 |
| 6 h | 26.55 | 4.93 | 18.59 | 27.14 | | | 0.59 | 2.23 |
| 12 h | | | | 2.31 | | | 2.31 | |
| **compound** | **phenol** | | | | | | | |
| 1 min | 82.76 | 27.18 | 32.85 | 48.47 | 5.51 | 11.36 | -34.29 | -41.43 |
| 3 min | 97.53 | 14.29 | 14.66 | 88.36 | 3.79 | 4.29 | -9.17 | -9.40 |
| 5 min | 97.53 | 9.75 | 10.00 | 94.39 | 5.15 | 5.45 | -3.13 | -3.21 |
| 10 min | 94.59 | 6.17 | 6.52 | 94.40 | 6.87 | 7.28 | -0.19 | -0.20 |
| 30 min | 94.52 | 7.41 | 7.84 | 77.35 | 6.85 | 8.86 | -17.17 | -18.16 |
| 1 h | 66.15 | 7.27 | 10.99 | 61.48 | 5.00 | 8.13 | -4.67 | -7.06 |
| 2 h | 46.62 | 1.45 | 3.11 | 43.22 | 4.08 | 9.43 | -3.41 | -7.31 |
| 3 h | 32.83 | 3.89 | 11.87 | 31.74 | 2.56 | 8.06 | -1.08 | -3.30 |
| 6 h | 20.19 | 2.86 | 14.17 | 14.51 | | | -5.68 | -28.13 |

| | | | | | | | | |
|---|---|---|---|---|---|---|---|---|
| 12 h | 5.86 | 0.39 | 6.72 | 3.37 | | | -2.49 | -42.49 |
| | | | | | | | | |
| **compound** | **isophorone** | | | | | | | |
| 1 min | 1105.18 | 232.41 | 21.03 | 860.26 | 81.36 | 9.46 | -244.91 | -22.16 |
| 3 min | 1227.56 | 169.08 | 13.77 | 1591.38 | 186.57 | 11.72 | 363.82 | 29.64 |
| 5 min | 1229.55 | 35.79 | 2.91 | 1628.51 | 191.07 | 11.73 | 398.96 | 32.45 |
| 10 min | 1118.96 | 90.69 | 8.11 | 1454.30 | 150.64 | 10.36 | 335.35 | 29.97 |
| 30 min | 1006.77 | 182.75 | 18.15 | 1086.56 | 125.65 | 11.56 | 79.79 | 7.93 |
| 1 h | 748.49 | 160.05 | 21.38 | 849.61 | 82.30 | 9.69 | 101.12 | 13.51 |
| 2 h | 581.60 | 67.05 | 11.53 | 629.88 | 64.11 | 10.18 | 48.27 | 8.30 |
| 3 h | 514.24 | 70.79 | 13.77 | 514.04 | 54.39 | 10.58 | -0.20 | -0.04 |
| 6 h | 353.56 | 23.58 | 6.67 | 330.24 | | | -23.31 | -6.59 |
| 12 h | 186.75 | 18.84 | 10.09 | 145.70 | | | -41.05 | -21.98 |
| | | | | | | | | |
| **compound** | **naphthalene** | | | | | | | |
| 1 min | 131.95 | 32.10 | 24.33 | 61.21 | 2.26 | 3.69 | -70.74 | -53.61 |
| 3 min | 156.93 | 19.29 | 12.29 | 104.42 | 11.31 | 10.83 | -52.51 | -33.46 |
| 5 min | 154.79 | 13.48 | 8.71 | 108.67 | 11.90 | 10.95 | -46.13 | -29.80 |
| 10 min | 137.91 | 2.82 | 2.04 | 100.59 | 10.91 | 10.85 | -37.33 | -27.07 |
| 30 min | 118.14 | 5.20 | 4.40 | 83.66 | 9.92 | 11.86 | -34.48 | -29.19 |
| 1 h | 87.12 | 4.31 | 4.95 | 69.37 | 7.39 | 10.66 | -17.75 | -20.37 |
| 2 h | 62.56 | 4.21 | 6.72 | 51.90 | 5.77 | 11.11 | -10.65 | -17.03 |
| 3 h | 44.92 | 9.97 | 22.19 | 40.94 | 4.62 | 11.28 | -3.98 | -8.86 |
| 6 h | 29.41 | 1.64 | 5.56 | 21.73 | | | -7.68 | -26.11 |
| 12 h | 10.83 | 0.15 | 1.41 | 6.54 | | | -4.30 | -39.67 |
| **Skipping rope** | | | | | | | | |
| **compound** | **phenol** | | | | | | | |
| 1 min | 56.36 | 13.75 | 24.40 | 24.64 | 3.76 | 15.27 | -31.72 | -56.28 |
| 3 min | 55.08 | 8.39 | 15.24 | 37.09 | 1.49 | 4.02 | -17.99 | -32.66 |
| 5 min | 53.92 | 6.75 | 12.51 | 39.22 | 0.92 | 2.36 | -14.70 | -27.27 |
| 10 min | 55.32 | 6.07 | 10.98 | 42.20 | 0.77 | 1.82 | -13.12 | -23.72 |
| 30 min | 50.30 | 8.92 | 17.73 | 39.10 | 0.79 | 2.01 | -11.20 | -22.26 |
| 1 h | 39.55 | 2.58 | 6.53 | 33.75 | 0.91 | 2.70 | -5.80 | -14.66 |
| 2 h | 34.20 | 3.92 | 11.45 | 28.21 | 0.98 | 3.49 | -5.99 | -17.51 |
| 3 h | 27.15 | 1.46 | 5.39 | 24.67 | 0.57 | 2.32 | -2.48 | -9.13 |
| 6 h | 13.67 | 1.96 | 14.33 | 19.00 | | | 5.33 | 39.01 |
| 12 h | 15.17 | 1.25 | 8.25 | 14.87 | | | -0.30 | -1.98 |

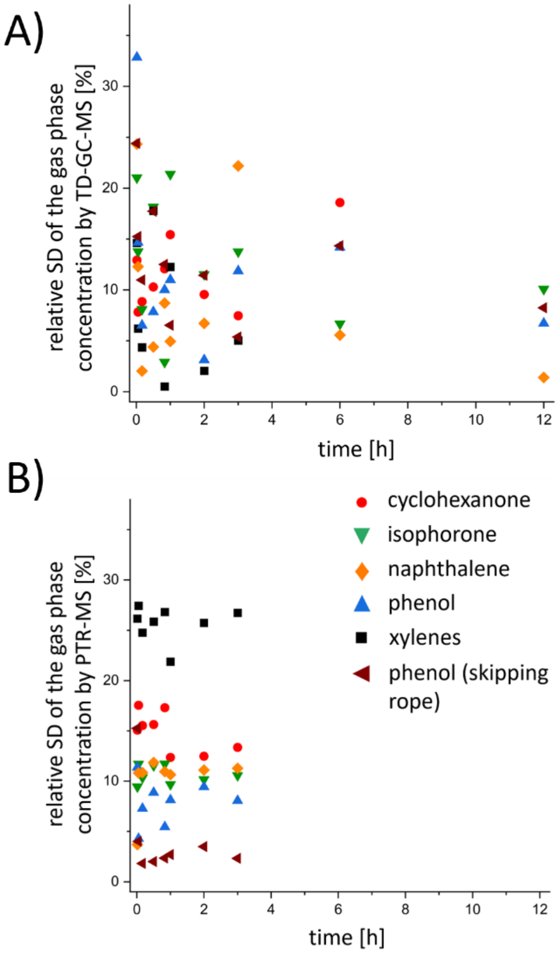


**Figure S2.** Relative SDs of compound concentrations from the poncho and skipping rope at different times as measured by A) TD-GC-MS and B) PTR-MS.

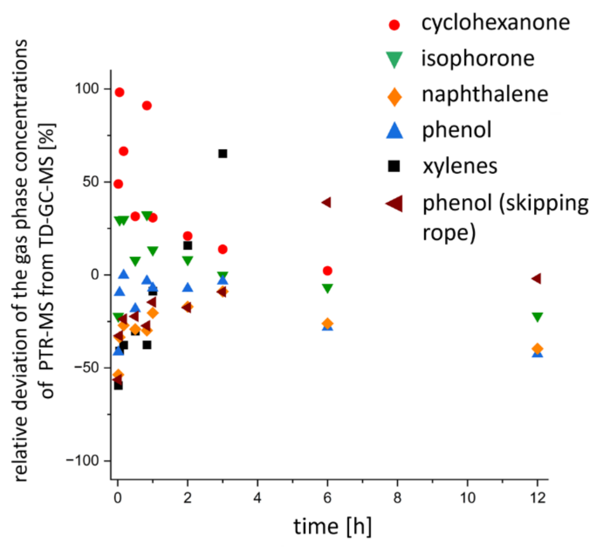


**Figure S3.** Relative deviations between the PTR-MS and TD-GC-MS analyses of the gas-phase concentrations of compounds emitted from the poncho and skipping rope over time.

**Table S6**. Comparison of the maximum concentrations $c_{max}$ [µg m$^{-3}$] and their relative deviations between the PTR-MS and the TD-GC-MS analyses.

| **compound** | **maximum concentration $c_{max}$ of emitted compounds in the chamber air [µg m$^{-3}$]** | | **relative deviation of $c_{max}$ from PTR-MS vs. TD-GC-MS analysis [%]** |
|---|---|---|---|
| | **TD-GC-MS** | **PTR-MS** | |
| **Poncho** | | | |
| sum of xylenes | 103.4 | 54.5 | -47.3 |
| cyclohexanone | 552.5 | 1082.4 | +95.9 |
| phenol | 97.5 | 94.5 | -3.1 |
| isophorone | 1229.5 | 1637.3 | +33.2 |
| naphthalene | 156.9 | 108.4 | -30.9 |
| **Skipping rope:** | | | |
| phenol | 56.4 | 42.2 | -25.1 |

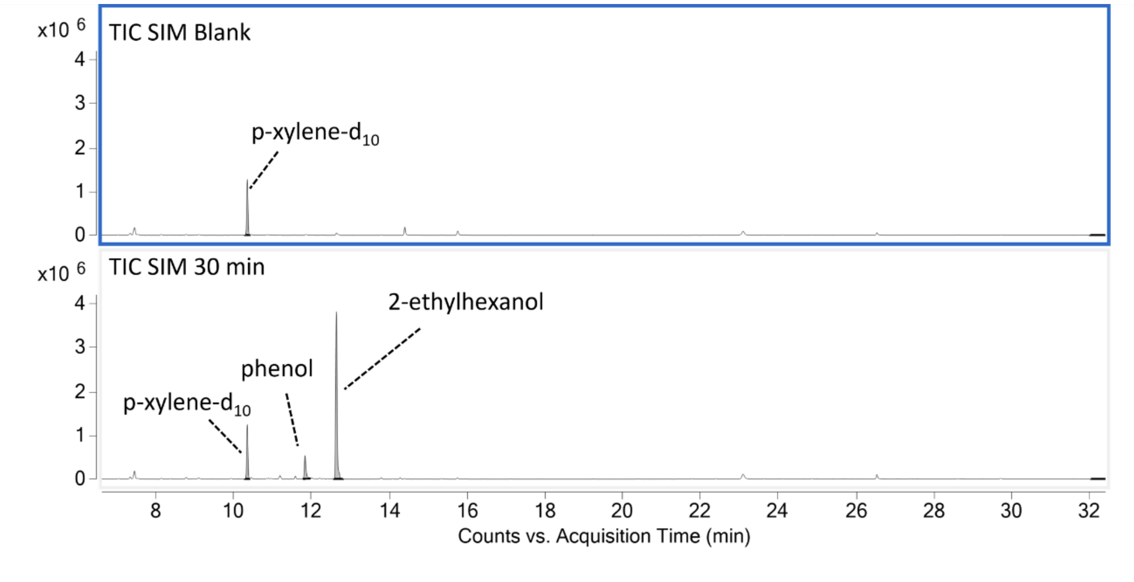


**Figure S4.** TD-GC-MS chromatograms in selected ion monitoring (SIM) mode (including qualifier and quantifier ions of all depicted analytes in the total ion chromatogram (TIC)) of the micro-chamber blank with the internal standard standard *p*-xylene-d$_{10}$ at $t_R$ = 10.3 min (top) and the skipping rope sample after 30 min in the micro-chamber (bottom).

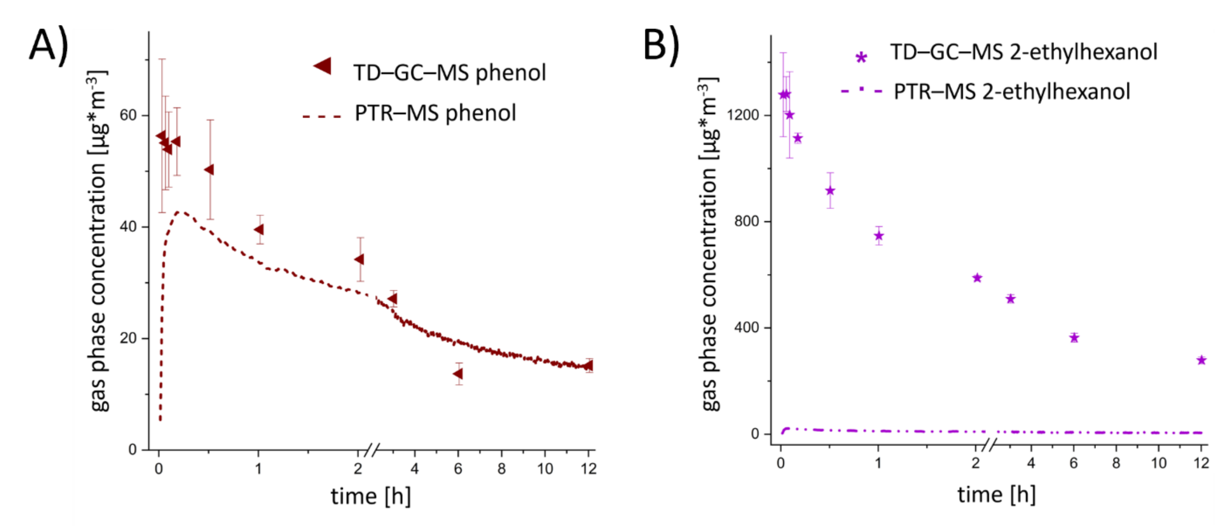


**Figure S5.** Emission profiles of A) phenol and B) 2-ethylhexanol from the skipping rope sample in a micro-chamber over 12 h, as determined by TD-GC-MS and PTR-MS analysis.

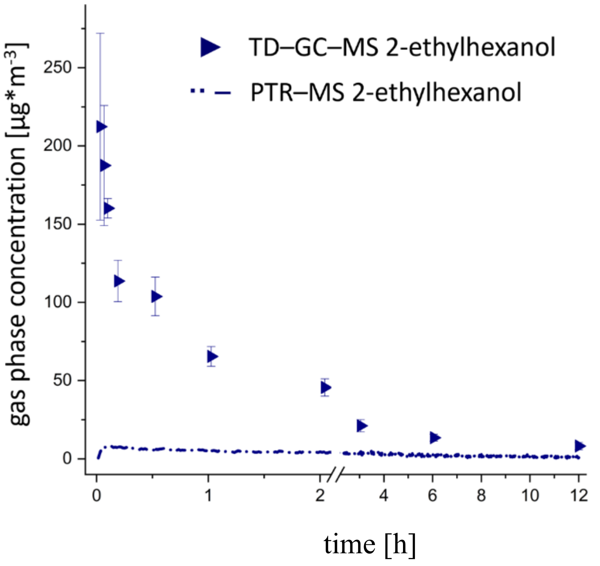


**Figure S6.** Emission profiles of 2-ethylhexanol from the poncho sample in a micro-chamber over 12 h, as determined by TD-GC-MS and PTR-MS analysis.

**Table S7.** Total quantities of emitted compounds (area under the emission rate curve – *$AUC_{ER}$*) [µg] and deviations between concentrations from PTR-MS and TD-GC-MS analysis.

| sampling time | *$AUC_{ER}$* TD-GC-MS [µg] | *$AUC_{ER}$* PTR-MS [µg] | deviation of *$AUC_{ER}$* from PTR-MS vs. TD-GC-MS analysis [µg] | relative deviation of *$AUC_{ER}$* from PTR-MS vs. TD-GC-MS analysis [%] |
|---|---|---|---|---|
| **Poncho** | | | | |
| **compound** | **xylenes** | | | |
| 1 min | - | - | - | - |
| 3 min | 0.020 | 0.012 | -0.008 | -39.9 |
| 5 min | 0.037 | 0.022 | -0.016 | -42.2 |
| 10 min | 0.072 | 0.041 | -0.032 | -44.0 |
| 30 min | 0.167 | 0.096 | -0.071 | -42.5 |
| 1 h | 0.243 | 0.150 | -0.093 | -38.2 |
| 2 h | 0.317 | 0.218 | -0.099 | -31.3 |
| 3 h | 0.346 | 0.255 | -0.091 | -26.4 |
| 6 h | | 0.316 | 0.316 | |
| 12 h | | 0.346 | 0.346 | |
| **compound** | **cyclohexanone** | | | |
| 1 min | - | - | - | - |
| 3 min | 0.110 | 0.143 | 0.033 | 29.6 |
| 5 min | 0.215 | 0.358 | 0.143 | 66.3 |
| 10 min | 0.441 | 0.785 | 0.343 | 77.8 |
| 30 min | 1.152 | 1.810 | 0.658 | 57.2 |
| 1 h | 1.900 | 2.754 | 0.854 | 44.9 |
| 2 h | 2.867 | 3.946 | 1.079 | 37.6 |
| 3 h | 3.486 | 4.665 | 1.179 | 33.8 |
| 6 h | 4.466 | 5.696 | 1.230 | 27.5 |
| 12 h | | 6.054 | 6.054 | |
| **compound** | **phenol** | | | |
| 1 min | - | - | - | - |
| 3 min | 0.018 | 0.009 | -0.009 | -50.3 |
| 5 min | 0.038 | 0.026 | -0.011 | -30.0 |
| 10 min | 0.086 | 0.073 | -0.012 | -14.1 |
| 30 min | 0.275 | 0.245 | -0.030 | -10.7 |
| 1 h | 0.516 | 0.449 | -0.067 | -13.0 |
| 2 h | 0.857 | 0.760 | -0.097 | -11.4 |
| 3 h | 1.095 | 0.984 | -0.111 | -10.1 |
| 6 h | 1.574 | 1.383 | -0.191 | -12.1 |
| 12 h | 2.043 | 1.647 | -0.395 | -19.4 |

| | | | | |
|---|---|---|---|---|
| **compound** | **isophorone** | | | |
| 1 min | - | - | - | - |
| 3 min | 0.233 | 0.160 | -0.073 | -31.4 |
| 5 min | 0.479 | 0.470 | -0.009 | -1.8 |
| 10 min | 1.066 | 1.257 | 0.191 | 17.9 |
| 30 min | 3.192 | 3.763 | 0.572 | 17.9 |
| 1 h | 5.825 | 6.600 | 0.776 | 13.3 |
| 2 h | 9.815 | 10.966 | 1.151 | 11.7 |
| 3 h | 13.103 | 14.398 | 1.295 | 9.9 |
| 6 h | 20.913 | 22.016 | 1.104 | 5.3 |
| 12 h | 30.625 | 30.056 | -0.568 | -1.9 |
| **compound** | **naphthalene** | | | |
| 1 min | - | - | - | - |
| 3 min | 0.029 | 0.011 | -0.018 | -61.3 |
| 5 min | 0.060 | 0.032 | -0.028 | -47.2 |
| 10 min | 0.133 | 0.085 | -0.048 | -36.3 |
| 30 min | 0.389 | 0.269 | -0.120 | -30.9 |
| 1 h | 0.697 | 0.495 | -0.203 | -29.1 |
| 2 h | 1.157 | 0.861 | -0.296 | -25.6 |
| 3 h | 1.474 | 1.135 | -0.339 | -23.0 |
| 6 h | 2.145 | 1.694 | -0.451 | -21.0 |
| 12 h | 2.870 | 2.154 | -0.716 | -24.9 |
| **Skipping rope** | | | | |
| **compound** | **phenol** | | | |
| 1 min | - | - | - | - |
| 3 min | 0.011 | 0.008 | -0.003 | -28.3 |
| 5 min | 0.022 | 0.016 | -0.006 | -28.9 |
| 10 min | 0.049 | 0.036 | -0.013 | -26.5 |
| 30 min | 0.155 | 0.119 | -0.036 | -23.5 |
| 1 h | 0.290 | 0.227 | -0.063 | -21.8 |
| 2 h | 0.513 | 0.411 | -0.102 | -19.9 |
| 3 h | 0.697 | 0.569 | -0.128 | -18.4 |
| 6 h | 1.066 | 0.956 | -0.110 | -10.3 |
| 12 h | 1.585 | 1.555 | -0.030 | -1.9 |

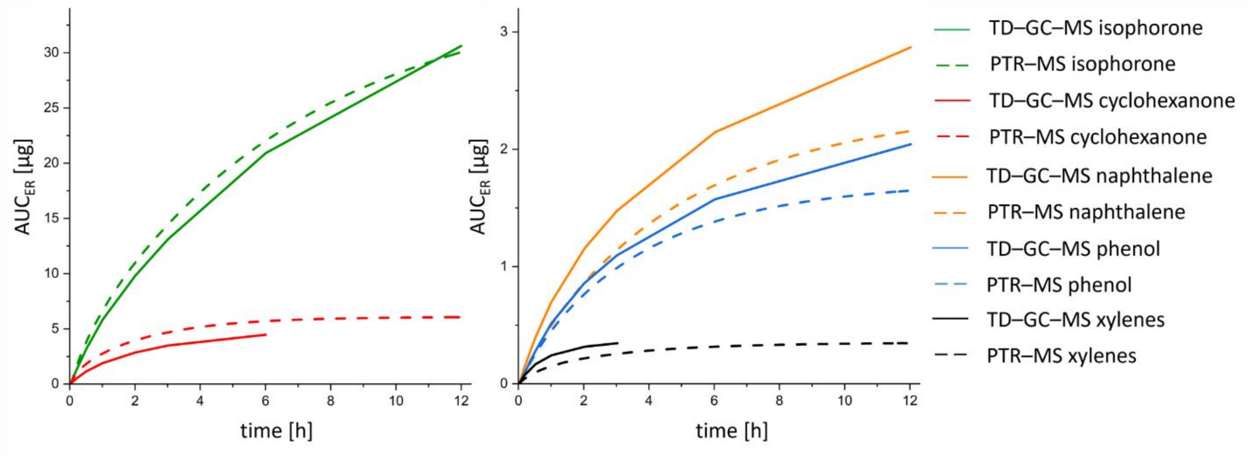


**Figure S7.** Total quantity of emitted compounds (area under the emission rate curve – *AUC$_{ER}$)* [µg] from the poncho in a micro-chamber over 12 h, as determined by PTR-MS and TD-GC-MS.
